# A Brief Review of Facial Emotion Recognition Based on Visual Information

**DOI:** 10.3390/s18020401

**Published:** 2018-01-30

**Authors:** Byoung Chul Ko

**Affiliations:** Department of Computer Engineering, Keimyung University, Daegu 42601, Korea; niceko@kmu.ac.kr; Tel.: +82-10-3559-4564

**Keywords:** facial emotion recognition, conventional FER, deep learning-based FER, convolutional neural networks, long short term memory, facial action coding system, facial action unit

## Abstract

Facial emotion recognition (FER) is an important topic in the fields of computer vision and artificial intelligence owing to its significant academic and commercial potential. Although FER can be conducted using multiple sensors, this review focuses on studies that exclusively use facial images, because visual expressions are one of the main information channels in interpersonal communication. This paper provides a brief review of researches in the field of FER conducted over the past decades. First, conventional FER approaches are described along with a summary of the representative categories of FER systems and their main algorithms. Deep-learning-based FER approaches using deep networks enabling “end-to-end” learning are then presented. This review also focuses on an up-to-date hybrid deep-learning approach combining a convolutional neural network (CNN) for the spatial features of an individual frame and long short-term memory (LSTM) for temporal features of consecutive frames. In the later part of this paper, a brief review of publicly available evaluation metrics is given, and a comparison with benchmark results, which are a standard for a quantitative comparison of FER researches, is described. This review can serve as a brief guidebook to newcomers in the field of FER, providing basic knowledge and a general understanding of the latest state-of-the-art studies, as well as to experienced researchers looking for productive directions for future work.

## 1. Introduction

Facial emotions are important factors in human communication that help us understand the intentions of others. In general, people infer the emotional states of other people, such as joy, sadness, and anger, using facial expressions and vocal tone. According to different surveys [1,2], verbal components convey one-third of human communication, and nonverbal components convey two-thirds. Among several nonverbal components, by carrying emotional meaning, facial expressions are one of the main information channels in interpersonal communication. Therefore, it is natural that research of facial emotion has been gaining lot of attention over the past decades with applications not only in the perceptual and cognitive sciences, but also in affective computing and computer animations [2].

Interest in automatic facial emotion recognition (FER) (Expanded form of the acronym FER is different in every paper, such as facial emotion recognition and facial expression recognition. In this paper, the term FER refers to facial emotion recognition as this study deals with the general aspects of recognition of facial emotion expression.) has also been increasing recently with the rapid development of artificial intelligent techniques, including in human-computer interaction (HCI) [3,4], virtual reality (VR) [5], augment reality (AR) [6], advanced driver assistant systems (ADASs) [7], and entertainment [8,9]. Although various sensors such as an electromyograph (EMG), electrocardiogram (ECG), electroencephalograph (EEG), and camera can be used for FER inputs, a camera is the most promising type of sensor because it provides the most informative clues for FER and does not need to be worn.

This paper first divides researches on automatic FER into two groups according to whether the features are handcrafted or generated through the output of a deep neural network. 

**Figure 1:**
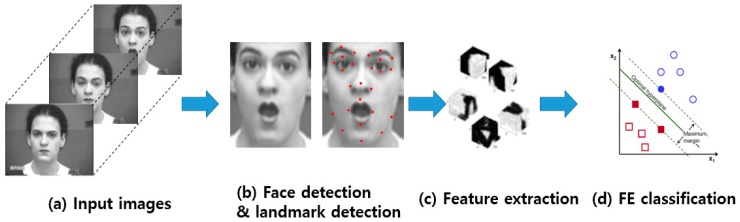
Procedure used in conventional FER approaches: From input images (**a**), face region and facial landmarks are detected (**b**), spatial and temporal features are extracted from the face components and landmarks (**c**), and the facial expression is determined based on one of facial categories using pre-trained pattern classifiers (face images are taken from CK+ dataset [10]) (**d**).

In conventional FER approaches, the FER is composed of three major steps, as shown in Figure 1: (1) face and facial component detection, (2) feature extraction, and (3) expression classification. First, a face image is detected from an input image, and facial components (e.g., eyes and nose) or landmarks are detected from the face region. Second, various spatial and temporal features are extracted from the facial components. Third, the pre-trained FE classifiers, such as a support vector machine (SVM), AdaBoost, and random forest, produce the recognition results using the extracted features.

In contrast to traditional approaches using handcrafted features, deep learning has emerged as a general approach to machine learning, yielding state-of-the-art results in many computer vision studies with the availability of big data [11].

**Figure 2:**
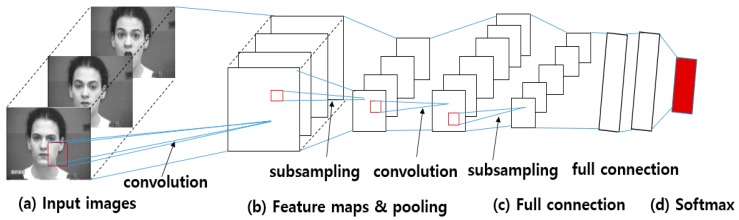
Procedure of CNN-based FER approaches: (**a**) The input images are convolved using filters in the convolution layers. (**b**) From the convolution results, feature maps are constructed and max-pooling (subsampling) layers lower the spatial resolution of the given feature maps. (**c**) CNNs apply fully connected neural-network layers behind the convolutional layers, and (**d**) a single face expression is recognized based on the output of softmax (face images are taken from CK+ dataset [10]).

Deep-learning-based FER approaches highly reduce the dependence on face-physics-based models and other pre-processing techniques by enabling “end-to-end” learning to occur in the pipeline directly from the input images [12]. Among the several deep-learning models available, the convolutional neural network (CNN), a particular type of deep learning, is the most popular network model. In CNN-based approaches, the input image is convolved through a filter collection in the convolution layers to produce a feature map. Each feature map is then combined to fully connected networks, and the face expression is recognized as belonging to a particular class-based the output of the softmax algorithm. Figure 2 shows the procedure used by CNN-based FER approaches.

FER can also be divided into two groups according to whether it uses frame or video images [13]. First, static (frame-based) FER relies solely on static facial features obtained by extracting handcrafted features from selected peak expression frames of image sequences. Second, dynamic (video-based) FER utilizes spatio-temporal features to capture the expression dynamics in facial expression sequences. Although dynamic FER is known to have a higher recognition rate than static FER because it provides additional temporal information, it does suffer from a few drawbacks. For example, the extracted dynamic features have different transition durations and different feature characteristics of the facial expression depending on the particular faces. Moreover, temporal normalization used to obtain expression sequences with a fixed number of frames may result in a loss of temporal scale information.

### 1.1. Terminology

**Figure 3:**
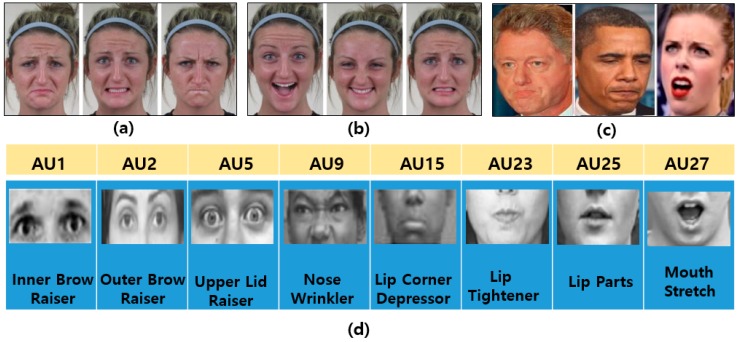
Sample examples of various facial emotions and AUs: (**a**) basic emotions (sad, fearful, and angry), (face images are taken from CE dataset [17]) (**b**) compound emotions (happily surprised, happily disgusted, and sadly fearful) (face images are taken from CE dataset [17]), (**c**) spontaneous expressions, and (face images are taken from YouTube) (**d**) AUs (upper and lower face) (face images are taken from CK+ dataset [10]).

Before reviewing researches related to FER, special terminology playing an important role in FER research is listed below:

- The facial action coding system (FACS) is a system based on facial muscle changes and can characterize facial actions to express individual human emotions as defined by Ekman and Friesen [14] in 1978. FACS encodes the movements of specific facial muscles called action units (AUs), which reflect distinct momentary changes in facial appearance [15].
- Facial landmarks (FLs) are visually salient points in facial regions such as the end of the nose, ends of the eye brows, and the mouth, as described in Figure 1b. The pairwise positions of each of two landmark points, or the local texture of a landmark, are used as a feature vector of FER. In general, FL detection approaches can be categorized into three types according to the generation of models such as active shape-based model (ASM) and appearance-based model (AAM), a regression-based model with a combination of local and global models, and CNN-based methods. FL models are trained model from the appearance and shape variations from a coarse initialization. Then, the initial shape is moved to a better position step-by-step until convergence [16].
- Basic emotions (BEs) are seven basic human emotions: happiness, surprise, anger, sadness, fear, disgust, and neutral, as shown in Figure 3a.
- Compound emotions (CEs) are a combination of two basic emotions. Du et al. [17] introduced 22 emotions, including seven basic emotions, 12 compound emotions most typically expressed by humans, and three additional emotions (appall, hate, and awe). Figure 3b shows some examples of CE.
- Micro expressions (MEs) indicate more spontaneous and subtle facial movements that occur involuntarily. They tend to reveal a person’s genuine and underlying emotions within a short period of time. Figure 3c shows some examples of MEs.
- Facial action units (AUs) code the fundamental actions (46 AUs) of individual or groups of muscles typically seen when producing the facial expressions of a particular emotion [17], as shown in Figure 3d. To recognize facial emotions, individual AU is detected and the system classify facial category according to the combination of AUs. For example, if an image has been annotated as having 1, 2, 25, and 26 AUs using an algorithm, the system will classify it as expressing an emotion of the “surprised” category, as indicated in Table 1.

Table 1 shows the prototypical AUs observed in each basic and compound emotion category.

### 1.2. Contributions of this Review

Despite the long history related to FER, there are no comprehensive literature reviews on the topic of FER. Some review papers [19,20] have focused solely on conventional researches without introducing deep-leaning-based approaches. Recently, Ghayoumi [21] introduced a quick review of deep learning in FER. However, only a review of simple differences between conventional approaches and deep-learning-based approaches was provided. Therefore, this paper is dedicated to a brief literature review, from conventional FER to recent advanced FER. The main contributions of this review are as follows:

- The focus is on providing a general understanding of the state-of-the art FER approaches, and helping new researchers understand the essential components and trends in the FER field.
- Various standard databases that include still images and video sequences for FER use are introduced, along with their purposes and characteristics.
- Key aspects are compared between conventional FER and deep-learning-based FER in terms of accuracy and resource requirements. Although deep-learning-based FER generally produces better FER accuracy than conventional FER, it also requires a large amount of processing capacity, such as a graphic processing unit (GPU) and central processing unit (CPU). Therefore, many current FER algorithms are still being used in embedded systems, including smartphones.
- A new direction and application for future FER studies are presented.

### 1.3. Organization of this Review

The remainder of this paper is organized as follows. In Section 2, conventional FER approaches are described along with a summary of the representative categories of FER systems and their main algorithms. In Section 3, advanced FER approaches using deep-learning algorithms are presented. In Section 4 and Section 5, a brief review of publicly available FER database and evaluation metrics with a comparison with benchmark results are provided. Finally, Section 6 offers some concluding remarks and discussion of future work.

## 2. Conventional FER Approaches 

For automatic FER systems, various types of conventional approaches have been studied. The commonality of these approaches is detecting the face region and extracting geometric features, appearance features, or a hybrid of geometric and appearance features on the target face. 

For the geometric features, the relationship between facial components is used to construct a feature vector for training [22,23]. Ghimire and Lee [23] used two types of geometric features based on the position and angle of 52 facial landmark points. First, the angle and Euclidean distance between each pair of landmarks within a frame are calculated, and second, the distance and angles are subtracted from the corresponding distance and angles in the first frame of the video sequence. For the classifier, two methods are presented, either using multi-class AdaBoost with dynamic time warping, or using a SVM on the boosted feature vectors.

The appearance features are usually extracted from the global face region [24] or different face regions containing different types of information [25,26]. As an example of using global features, Happy et al. [24] utilized a local binary pattern (LBP) histogram of different block sizes from a global face region as the feature vectors, and classified various facial expressions using a principal component analysis (PCA). Although this method is implemented in real time, the recognition accuracy tends to be degraded because it cannot reflect local variations of the facial components to the feature vector. Unlike a global-feature-based approach, different face regions have different levels of importance. For example, the eyes and mouth contain more information than the forehead and cheek. Ghimire et al. [27] extracted region-specific appearance features by dividing the entire face region into domain-specific local regions. Important local regions are determined using an incremental search approach, which results in a reduction of the feature dimensions and an improvement in the recognition accuracy. 

For hybrid features, some approaches [18,27] have combined geometric and appearance features to complement the weaknesses of the two approaches and provide even better results in certain cases. 

In video sequences, many systems [18,22,23,28] are used to measure the geometrical displacement of facial landmarks between the current frame and previous frame as temporal features, and extracts appearance features for the spatial features. The main difference between FER for still images and for video sequences is that the landmarks in the latter are tracked frame-by-frame and the system generates new dynamic features through displacement between the previous and current frames. Similar classification algorithms are then used in the video sequences, as described in Figure 1. To recognize micro-expression, high speed camera is used to capture video sequences of the face. Polikovsky et al. [29] presented facial micro-expressions recognition in video sequences captured from 200 frames per second (fps) high speed camera. This study divides face regions into specific regions, and then 3D-Gradients orientation histogram is generated from the motion in each region for FER.

Apart from FER of 2D images, 3D and 4D (dynamic 3D) recordings are increasingly used in expression analysis research because of the problems presented in 2D images caused by inherent variations in pose and illumination. 3D facial expression recognition generally consists of feature extraction and classification. One thing to note in 3D is that dynamic and static system are very different because of the nature of data. Static systems extract feature from statistical models such as deformable model, active shape model, analysis of 2D representations, and distance-based features. In contrast, dynamic systems utilize 3D image sequences for analysis of facial expressions such as 3D motion-based features. For FER, 3D images also use the similar conventional classification algorithms [29,30]. Although 3D-based FER showed higher performance than 2D-based FER, 3D and 4D-based FER also has certain problems such as a high computational cost owing to a high resolution and frame rate, as well as the amount of 3D information involved.

Some researchers [31,32,33,34,35] have tried to recognize facial emotions using infrared images instead of visible light spectrum (VIS) image because visible light (VIS) image is variable according to the status of illumination. Zhao et al. [31] used near-infrared (NIR) video sequences and LBP-TOP (Local binary patterns from three orthogonal planes) feature descriptors. This study uses component-based facial features to combine geometric and appearance information of face. For FER, a SVM and sparse representation classifiers are used. Shen et al. [32] used infrared thermal videos by extracting horizontal and vertical temperature difference from different facial sub-regions. For FER, the Adaboost algorithm with the weak classifiers of k-Nearest Neighbor is used. Szwoch and Pieniążek [33] recognized facial expression and emotion based only on depth channel from Microsoft Kinect sensor without using camera. This study uses local movements within the face area as the feature and recognized facial expressions using relations between particular emotions. Sujono and Gunawan [34] used Kinect motion sensor to detect face region based on depth information and active appearance model (AAM) to track the detected face. To role of AAM is to adjust shape and texture model in a new face, when there is variation of shape and texture comparing to the training result. To recognize facial emotion, the change of key features in AAM and fuzzy logic based on prior knowledge derived from FACS are used. Wei et al. [35] proposed FER using color and depth information by Kinect sensor together. This study extracts facial feature points vector by face tracking algorithm using captured sensor data and recognize six facial emotions by random forest algorithm. 

Commonly, conventional approaches determine features and classifiers by experts. For feature extraction, many well-known handcrafted feature, such as HoG, LBP, distance and angle relation between landmarks are used and the pre-trained classifiers, such as SVM, AdaBoost, and random forest, are also used for FE recognition based on the extracted features. Conventional approaches require relatively lower computing power and memory than deep learning-based approaches. Therefore, these approaches are still being studied for use in real-time embedded systems because of their low computational complexity and high degree of accuracy [22]. However, feature extraction and the classifiers should be designed by the programmer and they cannot be jointly optimized to improve performance [36,37]. 

Table 2 summarizes the representative conventional FER approaches and their main advantages.

## 3. Deep-Learning Based FER Approaches 

In recent decades, there has been a breakthrough in deep-learning algorithms applied to the field of computer vision, including a CNN and recurrent neural network (RNN). These deep-learning-based algorithms have been used for feature extraction, classification, and recognition tasks. The main advantage of a CNN is to completely remove or highly reduce the dependence on physics-based models and/or other pre-processing techniques by enabling “end-to-end” learning directly from input images [44]. For these reasons, CNN has achieved state-of-the-art results in various fields, including object recognition, face recognition, scene understanding, and FER.

A CNN contains three types of heterogeneous layers: convolution layer, max pooling layer, and fully connected layers, as shown in Figure 2. Convolutional layers take image or feature maps as the input, and convolve these inputs with a set of filter banks in a sliding-window manner to output feature maps that represent a spatial arrangement of the facial image. The weights of convolutional filters within a feature map are shared, and the inputs of the feature map layer are locally connected [45]. Second, subsampling layers lower the spatial resolution of the representation by averaging or max-pooling the given input feature maps to reduce their dimensions and thereby ignore variations in small shifts and geometric distortions [45,46]. The last fully connected layers of a CNN structure compute the class scores on the entire original image. Most deep-learning-based methods [46,47,48,49] have adapted a CNN directly for AU detection. 

Breuer and Kimmel [47] employed CNN visualization techniques to understand a model learned using various FER datasets, and demonstrated the capability of networks trained on emotion detection, across both datasets and various FER-related tasks. Jung et al. [48] used two different types of CNN: the first extracts temporal appearance features from the image sequences, whereas the second extracts temporal geometry features from temporal facial landmark points. These two models are combined using a new integration method to boost the performance of facial expression recognition. 

Zhao et al. [49] proposed deep region and multi-label learning (DRML), which is a unified deep network. DRML is a region layer that uses feed-forward functions to induce important facial regions, and forces the learned weights to capture structural information of the face. The complete network is end-to-end trainable, and automatically learns representations robust to variations inherent within a local region. 

**Figure 4:**
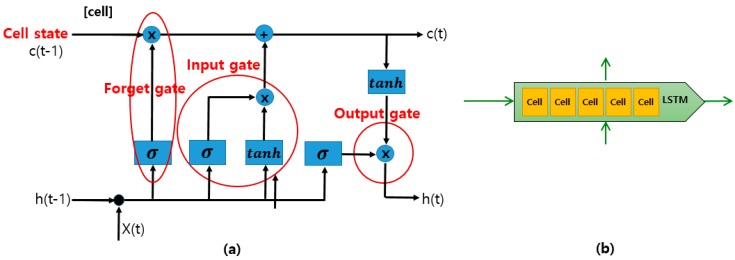
The basic structure of an LSTM, adapted from [50]. (**a**) One LSTM cell contains four interacting layers: the cell state, an input gate layer, a forget gate layer, and an output gate layer, (**b**) The repeating module of cells in an LSTM.

As we determined in our review, many approaches have adopted a CNN directly for FER use. However, because CNN-based methods cannot reflect temporal variations in the facial components, a recent hybrid approach combining a CNN for the spatial features of individual frames, and long short-term memory (LSTM) for the temporal features of consecutive frames, was developed. LSTM is a special type of RNN capable of learning long-term dependencies. LSTMs are explicitly designed to solve the long-term dependency problem using short-term memory. An LSTM has a chain-like structure, although the repeating modules have a different structure, as shown in Figure 4. All recurrent neural networks have a chain-like form of four repeating modules of a neural network [50]:

- The cell state is a horizontal line running through the top of the diagram, as shown in Figure 4. An LSTM has the ability to remove or add information to the cell state.
- A forget gate layer is used to decide what new information to store in the cell state.
- An input gate layer is used to decide which values will be updated in the cell.
- An output gate layer provides outputs based on the cell state.

The LSTM or RNN model for modeling sequential images has two advantages compared to standalone approaches. First, LSTM models are straightforward in terms of fine-tuning end-to-end when integrated with other models such as a CNN. Second, an LSTM supports both fixed-length and variable-length inputs or outputs [51]. 

The representative studies using a combination of a CNN and an LSTM (RNN) include the following:

Kahou et al. [11] proposed a hybrid RNN-CNN framework for propagating information over a sequence using a continuously valued hidden-layer representation. In this work, the authors presented a complete system for the 2015 Emotion Recognition in the Wild (EmotiW) Challenge [52], and proved that a hybrid CNN-RNN architecture for a facial expression analysis can outperform a previously applied CNN approach using temporal averaging for aggregation.

Kim et al. [13] utilized representative expression-states (e.g., the onset, apex, and offset of expressions), which can be specified in facial sequences regardless of the expression intensity. The spatial image characteristics of the representative expression-state frames are learned using a CNN. In the second part, temporal characteristics of the spatial feature representation in the first part are learned using an LSTM of the facial expression. 

Chu et al. [53] proposed a multi-level facial AU detection algorithm combining spatial and temporal features. First, the spatial representations are extracted using a CNN, which is able to reduce person-specific biases caused by handcrafted descriptors (e.g., HoG and Gabor). To model the temporal dependencies, LSTMs are stacked on top of these representations, regardless of the lengths of the input video sequences. The outputs of CNNs and LSTMs are further aggregated into a fusion network to produce a per-frame prediction of 12 AUs. 

Hasani and Mahoor [54] proposed the 3D Inception-ResNet architecture followed by an LSTM unit that together extracts the spatial relations and temporal relations within the facial images between different frames in a video sequence. Facial landmark points are also used as inputs of this network, emphasizing the importance of facial components rather than facial regions, which may not contribute significantly to generating facial expressions. 

Graves et al. [55] used a recurrent network to consider the temporal dependencies present in the image sequences during classification. In experimental results using two types of LSTM (bidirectional LSTM and unidirectional LSTM), this study proved that a bidirectional network provides a significantly better performance than a unidirectional LSTM. 

Jain et al. [56] proposed a multi-angle optimal pattern-based deep learning (MAOP-DL) method to rectify the problem of sudden changes in illumination, and find the proper alignment of the feature set by using multi-angle-based optimal configurations. Initially, this approach subtracts the background and isolates the foreground from the images, and then extracts the texture patterns and the relevant key features of the facial points. The relevant features are then selectively extracted, and an LSTM-CNN is employed to predict the required label for the facial expressions. 

Commonly, deep learning-based approaches determine features and classifiers by deep neural networks experts, unlike conventional approaches. Deep learning-based approaches extract optimal features with the desired characteristics directly from data using deep convolutional neural networks. However, it is not easy to collect a large amount of training data for the facial emotion under the different conditions enough to learn deep neural networks. Moreover, deep learning-based approaches require more a higher-level and massive computing device than convention approaches to operate training and testing [35]. Therefore, it is necessary to reduce the computational burden at inference time of deep learning algorithm.

Among the many approaches based on a standalone CNN or combination of LSTM and CNN, some representative works are shown in Table 3.

**Figure 5:**
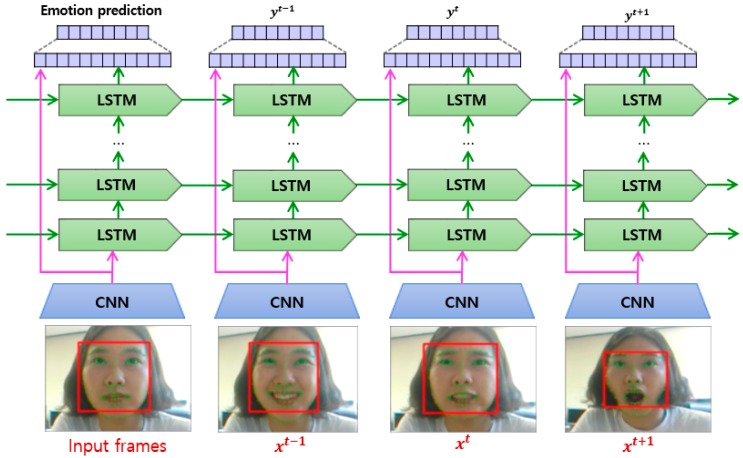
Overview of the general hybrid deep-learning framework for FER. The outputs of the CNNs and LSTMs are further aggregated into a fusion network to produce a per-frame prediction, adapted from [53].

As determined through our review conducted thus far, the general frameworks of the hybrid CNN-LSTM and CNN-RNN-based FER approaches have similar structures, as shown in Figure 5. In summary, the basic framework of CNN-LSTM (RNN) is to combine an LSTM with a deep hierarchical visual feature extractor such as a CNN model. Therefore, this hybrid model can learn to recognize and synthesize temporal dynamics for tasks involving sequential images. As shown in Figure 5, each visual feature determined through a CNN is passed to the corresponding LSTM, and produces a fixed or variable-length vector representation. The outputs are then passed into a recurrent sequence-learning module. Finally, the predicted distribution is computed by applying softmax [51,53].

## 4. Brief Introduction to FER Database 

In the field of FER, numerous databases have been used for comparative and extensive experiments. Traditionally, human facial emotions have been studied using either 2D static images or 2D video sequences. A 2D-based analysis has difficulty handling large pose variations and subtle facial behaviors. The analysis of 3D facial emotions will facilitate an examination of the fine structural changes inherent in spontaneous expressions [40]. Therefore, this sub-section briefly introduces some popular databases related to FER consisting of 2D and 3D video sequences and still images:

- The Extended Cohn-Kanade Dataset (CK+) [10]: CK+ contains 593 video sequences on both posed and non-posed (spontaneous) emotions, along with additional types of metadata. The age range of its 123 subjects is from 18 to 30 years, most of who are female. Image sequences may be analyzed for both action units and prototypic emotions. It provides protocols and baseline results for facial feature tracking, AUs, and emotion recognition. The images have pixel resolutions of 640 × 480 and 640 × 490 with 8-bit precision for gray-scale values.
- Compound Emotion (CE) [17]: CE contains 5060 images corresponding to 22 categories of basic and compound emotions for its 230 human subjects (130 females and 100 males, mean age of 23). Most ethnicities and races are included, including Caucasian, Asian, African, and Hispanic. Facial occlusions are minimized, with no glasses or facial hair. Male subjects were asked to shave their faces as cleanly as possible, and all participants were also asked to uncover their forehead to fully show their eyebrows. The photographs are color images taken using a Canon IXUS with a pixel resolution of 3000 × 4000.
- Denver Intensity of Spontaneous Facial Action Database (DISFA) [38]: DISFA consists of 130,000 stereo video frames at high resolution (1024 × 768) of 27 adult subjects (12 females and 15 males) with different ethnicities. The intensities of the AUs (0–5 scale) for all video frames were manually scored using two human experts in FACS. The database also includes 66 facial landmark points for each image in the database. The original size of each facial image is 1024 pixels × 768 pixels.
- Binghamton University 3D Facial Expression (BU-3DFE) [40]: Because 2D still images of faces are commonly used in FER, Yin et al. [40] at Binghamton University proposed a databases of annotated 3D facial expressions, namely, BU-3DFE 3D. It was designed for research on 3D human faces and facial expressions, and for the development of a general understanding of human behavior. It contains a total of 100 subjects, 56 females and 44 males, displaying six emotions. There are 25 3D facial emotion models per subject in the database, and a set of 83 manually annotated facial landmarks associated with each model. The original size of each facial image is 1040 pixels × 1329 pixels.
- Japanese Female Facial Expressions (JAFFE) [41]: The JAFFE database contains 213 images of seven facial emotions (six basic facial emotions and one neutral) posed by ten different female Japanese models. Each image was rated based on six emotional adjectives using 60 Japanese subjects. The original size of each facial image is 256 pixels × 256 pixels.
- Extended Yale B face (B+) [42]: This database consists of a set of 16,128 facial images taken under a single light source, and contains 28 distinct subjects for 576 viewing conditions, including nine poses for each of 64 illumination conditions. The original size of each facial image is 320 pixels × 243 pixels.
- MMI [43]: MMI consists of over 2900 video sequences and high-resolution still images of 75 subjects. It is fully annotated for the presence of AUs in the video sequences (event coding), and partially coded at the frame-level, indicating for each frame whether an AU is in a neutral, onset, apex, or offset phase. It contains a total of 238 video sequences on 28 subjects, both males and females. The original size of each facial image is 720 pixels × 576 pixels.
- Binghamton-Pittsburgh 3D Dynamic Spontaneous (BP4D-Spontanous) [58]: BP4D-spontanous is a 3D video database that includes a diverse group of 41 young adults (23 women, 18 men) with spontaneous facial expressions. The subjects were 18–29 years in age. Eleven are Asian, six are African-American, four are Hispanic, and 20 are Euro-Americans. The facial features were tracked in the 2D and 3D domains using both person-specific and generic approaches. The database promotes the exploration of 3D spatiotemporal features during subtle facial expressions for a better understanding of the relation between pose and motion dynamics in facial AUs, as well as a deeper understanding of naturally occurring facial actions. The original size of each facial image is 1040 pixels × 1329 pixels.
- The Karolinska Directed Emotional Face (KDEF) [59]: This database contains 4900 images of human emotional facial expressions. The database consists of 70 individuals, each displaying seven different emotional expressions photographed from five different angles. The original size of each facial image is 562 pixels × 762 pixels.

Table 4 shows a summary of these publicly available databases.

**Figure 6:**
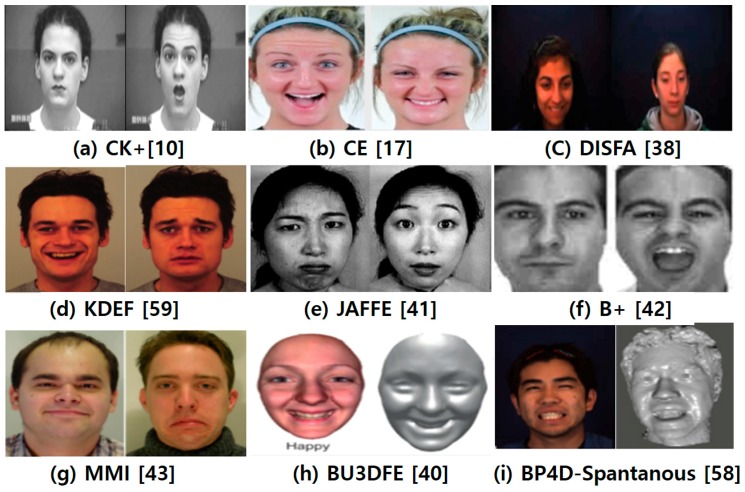
Examples of nine representative databases related to FER. Databases (**a**) through (**g**) support 2D still images and 2D video sequences, and databases (**h**) through (**i**) support 3D video sequences.

Figure 6 shows examples of the nine databases for FER with 2D and 3D images and video sequences.

Unlike the databases described above, MPI facial expression database [60] collects a large variety of natural emotional and conversational expressions under the assumption that people understand emotions by analyzing both the conversational expressions as well as the emotional expressions. This database consists of more than 18,800 samples of video sequences from 10 females and nine male models displaying various facial expressions recorded from one frontal and two lateral views. 

In recent, other sensors, such as NIR camera, thermal camera, and Kinect sensors, are having interesting of FER researches because visible light image is easily changeable when there are changes in environmental illumination conditions. As the database captured from NIR camera, Oulu-CASIA NIR&VIS facial expression database [31] consists of six expressions from 80 people between 23 and 58 years old. 73.8% of the subjects are males. Natural visible and infrared facial expression (USTC-NVIE) database [32] collected both spontaneous and posed expressions of more than 100 subjects simultaneously using a visible and an infrared thermal camera. Facial expressions and emotions database (FEEDB) is a multimodal database of facial expressions and emotion recorded using Microsoft Kinect sensor. It contains of 1650 recordings of 50 persons posing for 33 different facial expressions and emotions [33]. 

As described here, various sensors other than the camera sensor are used for FER, but there is a limitation in improving the recognition performance with only one sensor. Therefore, it is predicted that the attempts to increase the FER through the combination of various sensors, will continue in the future.

## 5. Performance Evaluation of FER

Given the FER approaches, evaluation metrics of the FER approaches are crucial because they provide a standard for a quantitative comparison. In this section, a brief review of publicly available evaluation metrics and a comparison with the benchmark results are provided.

### 5.1. Subject-Independent and Cross-Database Tasks

Many approaches are used to evaluate the accuracy using two different experiment protocols: subject-independent and cross-dataset tasks [55]. First, a subject-independent task splits each database into training and validation sets in a strict subject-independent manner. This task is also called a K-fold cross-validation. The purpose of K-fold cross-validation is to limit problems such as overfitting and provide insight regarding how the model will generalize into an independent unknown dataset [61]. With the K-fold cross-validation technique, each dataset is evenly partitioned into K folds with exclusive subjects. Then, a model is iteratively trained using K-1 folds and evaluated on the remaining fold, until all subjects are tested. Validation is conducted using almost less than 20% of the training subjects. The accuracy is estimated by averaging the recognition rate over K folds. For example, in ten-fold cross-validation adopted for an evaluation, nine folds are used for training, and one fold is used for testing. After this process is performed ten different times, the accuracies of the ten results are averaged and defined as the classifier performance.

The second protocol is a cross-database task. In this task, one dataset is used entirely for testing the model, and the remaining datasets listed in Table 4 are used to train the model. The model is iteratively trained using K-1 datasets and evaluated on the remaining dataset repeatedly until all datasets have been tested. The accuracy is estimated by averaging the recognition rate over K datasets in a manner similar to K-fold cross-validation.

### 5.2. Evaluation Metrics

The evaluation metrics of FER are classified into four methods using different attributes: precision, recall, accuracy, and F1-score.

The precision (P) is defined as TP/(TP + FP), and the recall (R) is defined as TP/(TP + FN), where TP is the number of true positives in the dataset, FN is the number of false negatives, and FP is the number of false positives. The precision is the fraction of automatic annotations of emotion *i* that are correctly recognized. The recall is the number of correct recognitions of emotion *i* over the actual number of images with emotion *i* [18]. The accuracy is the ratio of true outcomes (both true positive to true negative) to the total number of cases examined.

(1) $$\mathrm{Accuracy}\;\left(\mathrm{ACC}\right)=\frac{TP+TN}{Total\;population}$$

Another metric, the F1-score, is divided into two metrics depending on whether they use spatial or temporal data: frame-based F1-score (F1-frame) and event-based F1-score (F1-event). Each metric captures different properties of the results. This means that a frame-based F-score has predictive power in terms of spatial consistency, whereas an event-based F-score has predictive power in terms of the temporal consistency [62]. A frame-based F1-score is defined as

(2) $$F1-\mathrm{frame}=\frac{2RP}{R+P}$$

An event-based F1-score is used to measure the emotion recognition performance at the segment level because emotions occur as a temporal signal.

(3) $$F1-\mathrm{event}=\frac{2ER\times EP}{\left(ER+EP\right)}$$

where *ER* and *EP* are event-based recall and precision. *ER* is the ratio of correctly detected events over the true events, while the *EP* is the ratio of correctly detected events over the detected events. F1-event considers that there is an event agreement if the overlap is above a certain threshold [63].

### 5.3. Evaluation Results

To show a direct comparison between conventional handcrafted-feature-based approaches and deep-learning-based approaches, this review lists public results on the MMI dataset. Table 5 shows the comparative recognition rate of six conventional approaches and six deep-learning-based approaches.

As shown in Table 5, deep-learning-based approaches outperform conventional approaches with an average of 72.65% versus 63.2%. In conventional FER approaches, the reference [68] has the highest performance than other algorithms. This study tried to compute difference information between the peak expression face and its intra class variation in order to reduce the effect of the facial identity in the feature extraction. Because the feature extraction is robust to face rotation and misalignment, this study achieves relatively accurate FER than other conventional methods. Among several deep-learning-based approaches, two have a relatively higher performance compared to several state-of-the-art methods; a complex CNN network proposed in [72] consists of two convolutional layers, each followed by max pooling and four Inception layers. This network has a single-component architecture that takes registered facial images as the input and classifies them into one of six basic or one neutral expression. The highest performance approach [13] also consists of two parts. In the first part, the spatial image characteristics of the representative expression-state frames are learned using a CNN. In the second part, the temporal characteristics of the spatial feature representation in the first part are learned using an LSTM of the facial expression. Based on the accuracy of a complex hybrid approach using spatio-temporal feature representation learning, the FER performance of largely affected not only by the spatial changes but also by the temporal changes.

Although deep-learning-based FER approaches have achieved great success in experimental evaluations, a number of issues remain that deserve further investigation:

- A large-scale dataset and massive computing power are required for training as the structure becomes increasingly deep.
- Large numbers of manually collected and labeled datasets are needed.
- Large memory is demanded, and the training and testing are both time consuming. These memories demanding and computational complexities make deep learning ill-suited for deployment on mobile platforms with limited resources [73].
- Considerable skill and experience are required to select suitable hyper parameters, such as the learning rate, kernel sizes of the convolutional filters, and the number of layers. These hyper-parameters have internal dependencies that make them particularly expensive for tuning.
- Although they work quite well for various applications, a solid theory of CNNs is still lacking, and thus users essentially do not know why or how they work.

## 6. Conclusions

This paper presented a brief review of FER approaches. As we described, such approaches can be divided into two main streams: conventional FER approaches consisting of three steps, namely, face and facial component detection, feature extraction, and expression classification. The classification algorithms used in conventional FER include SVM, Adaboost, and random forest; by contrast, deep-learning-based FER approaches highly reduce the dependence on face-physics-based models and other pre-processing techniques by enabling “end-to-end” learning in the pipeline directly from the input images. As a particular type of deep learning, a CNN visualizes the input images to help understand the model learned through various FER datasets, and demonstrates the capability of networks trained on emotion detection, across both the datasets and various FER related tasks. However, because CNN-based FER methods cannot reflect the temporal variations in the facial components, hybrid approaches have been proposed by combining a CNN for the spatial features of individual frames, and an LSTM for the temporal features of consecutive frames. A few recent studies have provided an analysis of a hybrid CNN-LSTM (RNN) architecture for facial expressions that can outperform previously applied CNN approaches using temporal averaging for aggregation. However, deep-learning-based FER approaches still have a number of limitations, including the need for large-scale datasets, massive computing power, and large amounts of memory, and are time consuming for both the training and testing phases. Moreover, although a hybrid architecture has shown a superior performance, micro-expressions remain a challenging task to solve because they are more spontaneous and subtle facial movements that occur involuntarily. 

This paper also briefly introduced some popular databases related to FER consisting of both video sequences and still images. In a traditional dataset, human facial expressions have been studied using either static 2D images or 2D video sequences. However, because a 2D-based analysis has difficulty handling large variations in pose and subtle facial behaviors, recent datasets have considered 3D facial expressions to better facilitate an examination of the fine structural changes inherent to spontaneous expressions. 

Furthermore, evaluation metrics of FER-based approaches were introduced to provide standard metrics for comparison. Evaluation metrics have been widely evaluated in the field of recognition, and precision and recall are mainly used. However, a new evaluation method for recognizing consecutive facial expressions, or applying micro-expression recognition for moving images, should be proposed. 

Although studies on FER have been conducted over the past decade, in recent years the performance of FER has been significantly improved through a combination of deep-learning algorithms. Because FER is an important way to infuse emotion into machines, it is advantageous that various studies on its future application are being conducted. If emotional oriented deep-learning algorithms can be developed and combined with additional Internet-of-Things sensors in the future, it is expected that FER can improve its current recognition rate, including even spontaneous micro-expressions, to the same level as human beings.

## Figures and Tables

**Table 1:** Prototypical AUs observed in each basic and compound emotion category, adapted from [18].

Category	AUs	Category	AUs
Happy	12, 25	Sadly disgusted	4, 10
Sad	4, 15	Fearfully angry	4, 20, 25
Fearful	1, 4, 20, 25	Fearfully surprised	1, 2, 5, 20, 25
Angry	4, 7, 24	Fearfully disgusted	1, 4, 10, 20, 25
Surprised	1, 2 , 25, 26	Angrily surprised	4, 25, 26
Disgusted	9, 10, 17	Disgusted surprised	1, 2, 5, 10
Happily sad	4, 6, 12, 25	Happily fearful	1, 2, 12, 25, 26
Happily surprised	1, 2, 12, 25	Angrily disgusted	4, 10, 17
Happily disgusted	10, 12, 25	Awed	1, 2, 5, 25
Sadly fearful	1, 4, 15, 25	Appalled	4, 9, 10
Sadly angry	4, 7, 15	Hatred	4, 7, 10
Sadly surprised	1, 4, 25, 26	-	-

**Table 2:** A summary of publicly available databases related to FER. (The detail information on database is described in Section 4).

Reference	Emotions Analyzed	Visual Features	Decision Methods	Database
Compound emotion [17]	Seven emotions and 22 compound emotions	Distribution between each pair of fiducials Appearance defined by Gabor filters	Nearest-mean classifier, Kernel subclass discriminant analysis	CE [17]
EmotioNet [18]	23 basic and compound emotions	Euclidean distances between normalized landmarks Angles between landmarks Gabor filters centered at of the landmark points	Kernel subclass discriminant analysis	CE [17] CK+ [10], DISFA [38],
Real-time mobile [22]	Seven emotions	Active shape model fitting landmarks Displacement between landmarks	SVM	CK+ [10]
Ghimire and Lee [23]	Seven emotions	Displacement between landmarks in continuous frames	Multi-class AdaBoost, SVM	CK+ [10]
Global Feature [24]	Six emotions	Local binary pattern (LBP) histogram of a face image	Principal component analysis (PCA)	Self-generated
Local region specific feature [33]	Seven emotions	Appearance of LBP features from specific local regions Geometric normalized central moment features from specific local regions.	SVM	CK+ [10]
InfraFace [34]	Seven emotions, 17 AUs detected	Histogram of gradients (HoG)	A linear SVM	CK+ [10]
3D facial expression [39]	Six prototypical emotions	3D curve shape and 3D patch shape by analyzing shapes of curves to the shapes of patches	Multiboosting and SVM	BU-3DFE [40]
Stepwise approach [31]	Six prototypical emotions	Stepwise linear discriminant analysis (SWLDA) used to select the localized features from the expression	Hidden conditional random fields (HCRFs)	CK+ [10], JAFFE [41], B+ [42], MMI [43]

**Table 3:** Summary of FER systems based on deep learning.

Reference	Emotions Analyzed	Recognition Algorithm	Database
hybrid CNN-RNN [11]	Seven emotions	Hybrid RNN-CNN framework for propagating information over a sequence Using temporal averaging for aggregation	EmotiW [52]
Kim et al. [13]	Six emotions	Spatial image characteristics of the representative expression-state frames are learned using a CNN Temporal characteristics of the spatial feature representation in the first part are learned using an LSTM	MMI [43], CASME II [57]
Breuer and Kimmel [47]	Eight emotions, 50 AU detection	CNN-based feature extraction and inference	CK+ [10], NovaEmotions [47]
Joint Fine-Tunning [48]	Seven emotions	Two different models CNN for temporal appearance features CNN for temporal geometry features from temporal facial landmark points	CK+ [10], MMI [43]
DRML [49]	12 AUs for BP4D, eight AUs for DISFA	Feed-forward functions to induce important facial regions Learning of weights to capture structural information of the face	DISFA [38], BP4D [58]
Multi-level AU [53]	12 AU detection	Spatial representations are extracted by a CNN LSTMs for temporal dependencies	BP4D [58]
3D Inception-ResNet [54]	23 basic and compound emotions	LSTM unit that together extracts the spatial relations and temporal relations within facial images Facial landmark points are also used as inputs to this network	CK+ [10], DISFA [38]
Candide-3 [55]	Six emotions	Conjunction with a learned objective function for face model fitting Using a recurrent network for temporal dependencies present in the image sequences during classification.	CK+ [10]
Multi-angle FER [56]	Six emotions	Extraction of the texture patterns and the relevant key features of the facial points. Employment of LSTM-CNN to predict the required label for the facial expressions	CK+ [10], MMI [43]

**Table 4:** A summary of publicly available databases related to FER.

Database	Data Configuration	Web Link
CK+ [10]	593 video sequences on both posed and non-posed (spontaneous) emotions 123 subjects from 18 to 30 years in age Provides protocols and baseline results for facial feature tracking, action units, and emotion recognition Image resolutions of 640 × 480, and 640 × 490	http://www.consortium.ri.cmu.edu/ckagree/
CE [17]	5060 images corresponding to 22 categories of basic and compound emotions 230 human subjects (130 females and 100 males, mean age 23) Includes most ethnicities and races Image resolution of 3000 × 4000	http://cbcsl.ece.ohio-state.edu/dbform_compound.html
DISFA [38]	130,000 stereo video frames at high resolution 27 adult subjects (12 females and 15 males) 66 facial landmark points for each image Image resolution of 1024 × 768	http://www.engr.du.edu/mmahoor/DISFA.htm
BU-3DFE [40]	3D human faces and facial emotions 100 subjects in the database, 56 females and 44 males, with about six emotions 25 3D facial emotion models per subject Image resolution of 1040 × 1329	http://www.cs.binghamton.edu/~lijun/Research/3DFE/3DFE_Analysis.html
JAFFE [41]	213 images of seven facial emotions Ten different female Japanese models Six emotion adjectives by 60 Japanese subjects Image resolution of 256 × 256	http://www.kasrl.org/jaffe_info.html
B+ [42]	16,128 facial images 28 distinct subjects for 576 viewing conditions Image resolution of 320 × 243	http://vision.ucsd.edu/content/extended-yale-face-database-b-b
MMI [43]	Over 2900 video sequences and high-resolution still images of 75 subjects 238 video sequences on 28 subjects, male and female Image resolution of 720 × 576	https://mmifacedb.eu/
BP4D-Spontanous [58]	3D video database includes 41 participants (23 women, 18 men), with spontaneous facial emotions 11 Asians, six African-Americans, four Hispanics, and 20 Euro-Americans Image resolution of 1040 × 1329	http://www.cs.binghamton.edu/~lijun/Research/3DFE/3DFE_Analysis.html
KDEF [59]	4900 images of human facial expressions of emotion 70 individuals, seven different emotional expressions with 5 different angles Image resolution of 562 × 762	http://www.emotionlab.se/resources/kdef

**Table 5:** Recognition performance with MMI dataset, adapted from [11].

Type	Brief Description of Main Algorithms	Input	Accuracy (%)
Conventional (handcrafted-feature) FER approaches	Sparse representation classifier with LBP features [63]	Still frame	59.18
	Sparse representation classifier with local phase quantization features [64]	Still frame	62.72
	SVM with Gabor wavelet features [65]	Still frame	61.89
	Sparse representation classifier with LBP from three orthogonal planes [66]	Sequence	61.19
	Sparse representation classifier with local phase quantization feature from three orthogonal planes [67]	Sequence	64.11
	Collaborative expression representation CER [68]	Still frame	70.12
	**Average**		**63.20**
Deep-learning-based FER approaches	Deep learning of deformable facial action parts [69]	Sequence	63.40
	Joint fine-tuning in deep neural networks [48]	Sequence	70.24
	AU-aware deep networks [70]	Still frame	69.88
	AU-inspired deep networks [71]	Still frame	75.85
	Deeper CNN [72]	Still frame	77.90
	CNN + LSTM with spatio-temporal feature representation [13]	Sequence	78.61
	**Average**		**72.65**
